# Supplementary material for: Template models for simulation of surface manipulation of musculoskeletal extremities
Source: PLoS One. 2022 Aug 15;17(8):e0272051. doi: 10.1371/journal.pone.0272051 (PMC9377586; doi:10.1371/journal.pone.0272051)
Supplement: Attachment — Submitted filename: PlosOneAuthorResponse_secondresub.docx [file pone.0272051.s002.docx]

**Authors’ Responses to Questions and Comments:**

**All line numbers are indicated in the marked up document rather than the changes accepted copy.**

**Comments to the Author**
1. In response to my previous comment #8, you wrote that the simulation files were reduced to 9 points to save on repository space. However, I still don't understand why 6 out of these 9 points are without any contact (and force) so they are completely irrelevant for the parameter fitting. I think it is still not fully clear which of the experimental points (black dots in figure 5) were used as must points in FEBio, and which data was used for computing the objective function for the curve fitting. If the shared data contains only a few must points, can the reader reproduce the simulated results shown in figure 5?

**Author Response:**

The reviewer raised a valid point that the xplt files show 5 of the same data point, and so the .xplt files in the repository were cleaned. The .xplt file now shows only 5 points, with the initial state and then increments of 25% of the maximum simulation displacement value (25% at 1.25, 50% at 1.5, 75% at 1.75 and 100% prescribed displacement at time 2). The xplt values are merely samples of the simulation displacement which was prescribed in 1% increments. Note the simulation displacement is equal in direction, but proportionally greater than the experimental displacement magnitude, due to the cropping procedure utilized. The simulation data is fit to the experimental data by first removing all points below the initial experimental starting force. From this point, the displacement up until the maximum experimental displacement magnitude is captured in 1% increments. FEBio simulation extends beyond this point, since analysis of the displacements was only conducted after the simulation was finished. The results in figure 5 are reproducible, because the inverse FEA script does **not** parse the xplt file, rather the script parses the log file. The log file contains data from the 1% increments to fit the simulation data to the experimental data.

Changes were made in the demo example explained in the results to explain how the experimental data informs the cropping of the simulation data (line 220-224). The xplt file is not a 1:1 representation of the actual experiment, which I have now emphasized in the paper (lines 231-232). The captions for Figure 5 and 6 were also updated to emphasize that the simulation and xplt data are not a direct match of the experiment data, due to the fitting process.
